# Do Novel Biomarkers Have Utility in the Diagnosis and Prognosis of AKI?: Commentary

**DOI:** 10.34067/KID.0000000000000240

**Published:** 2023-12-28

**Authors:** Edward D. Siew

**Affiliations:** Division of Nephrology and Hypertension, Vanderbilt Center for Kidney Disease (VCKD) and Integrated Program for Acute Kidney Injury (AKI), Vanderbilt University Medical Center, Nashville, Tennessee and Tennessee Valley Health Systems (TVHS), Nashville Veterans Affairs Hospital, Tennessee

**Keywords:** acute renal failure

The past 20 years have witnessed growing recognition of AKI as an underappreciated consequence of acute illness. More granular creatinine and urine output AKI definitions have uncovered the significance of milder cases, short-term and long-term outcomes of AKI, and incorporation of creatinine changes as end points in clinical trials. Yet, phenotypic “blind spots” that can inform the onset, dynamics, and differing etiologies of this varied condition remain. The latter has motivated efforts to discover and validate novel biomarkers to fill these gaps.

In this issue of *Kidney360*, Drs. Kellum, Goldstein, and Coca debate the current diagnostic and prognostic usefulness of novel AKI biomarkers. Arguing the pro position, Drs. Kellum and Goldstein eloquently describe the known sensitivity and specificity limitations of serum creatinine while highlighting candidate markers that are detectable in the urine before changes in serum creatinine, provide additional prognostic information, and enrich for high-risk populations. They share their experiences using some biomarkers in routine practice and propose a needed framework for characterizing AKI that incorporates both functional and injury domains. Balancing this position, Dr. Coca argues that in addition to providing accurate and novel information, the bar for clinical utility of biomarkers should include demonstrating that the information gained can improve outcomes. He cautions of potential dangers in misinterpreting values, providing tangible examples of commonly encountered clinical scenarios, and suggests emerging areas, such as distinguishing between causes of AKI and long-term risk stratification.

The tension between early and late adopters of new tools is common in nephrology and medicine. Intrinsic to the optimism of early adopters is that the risk:benefit ratio for a given tool is likely to be favorable and improve the current standard of care. Admittedly, the limitations of the current battery of noninvasive diagnostic tools and lack of established pharmacotherapeutics to couple them with render the bar low. To date, most validation studies have used creatinine-based outcomes as a reference standard, the limitations of which for assessing parenchymal injury have been previously described.^1^ One study that used a histologic reference standard in a select population of deceased kidney donors indicated that biomarkers, such as neutrophil gelatinase–associated lipocalin, was superior to serum creatinine for diagnosing histologic tubular injury with higher specificity than conventional tools (*e.g.*, fractional excretion of sodium), although overall discrimination was modest.^2^ Nevertheless, the accumulated data demonstrating that altered biomarker levels strongly associate with developing AKI and AKI progression (as currently defined) and mortality have focused the framing of utility on the risk of experiencing these outcomes. The latter have begun to yield some examples of utility, such as enriching clinical trials with higher risk patients^3^ or helping to determine whether a rise in creatinine with aggressive control of BP is more likely to be a hemodynamic epiphenomenon rather than a reflection of tissue damage.^4^ Other natural extensions could include, but are not limited to, whether biomarkers can triage patients to higher or lower intensity care settings, refine diagnostic or resuscitation algorithms, or guide nephrotoxin stewardship more effectively.

Yet, as attempts to determine what optimal clinical responses to abnormal biomarker levels should be, it is also worth asking whether our view of utility needs to be broadened? Although risk stratification or more accurate indicators of damage are important, a critical concern is whether current biomarkers can improve phenotyping sufficiently to advance the translation of much-needed novel therapies? For example, enrollment in AKI trials often uses the clinical setting in which AKI occurs as a proxy for specific subtypes of AKI. Yet within these settings, AKI can remain heterogeneous. For example, data suggest that nephrotoxins can contribute to 20%–25% of AKI in hospitalized patients. Undiscoverable contamination of a trial designed to target a specific type of AKI (*e.g.*, sepsis-induced acute tubular necrosis) with a fraction of that amount could affect the ability to detect differences in outcomes if not accounted properly. Studies indicating that biomarkers can help distinguish specific subtypes of AKI, such as acute interstitial nephritis, from others, hold promise for reducing heterogeneity in clinical trials and better aligning study populations with intended biologic targets.^5^ In addition, efforts to creatively incorporate biomarker data with emerging techniques, such as machine learning, to identify novel phenotypes of AKI also hold promise for identifying treatment-responsive subphenotypes.^6^ Finally, well-funded efforts, such as the Kidney Precision Medicine Program,^7^ will also help determine how biomarkers can help reflect novel biologic targets.

In the interim, we need not await the arrival of novel therapeutics to meet the bar of actionable improvements in AKI. Indeed, evidence already exists that optimizing current care can improve outcomes.^3,8^ With numerous clinical uncertainties remaining in the management of AKI, including those suggested by Coca, carefully designed trials that test how biomarkers can guide or reflect management are needed to identify specific use cases, define the limits of interpretation, and quantify utility. Although such studies can and should be informed by anecdotal experience, the complexity and consequences of AKI warrant that our assessment of utility should not be limited to it. Finally, efforts to characterize the effect of AKI more fully on kidney health should be encouraged. For example, a recent study suggested that GFR may be significantly overestimated using creatinine compared with cystatin C in critically ill patients, resulting in inflated estimates of kidney function and recovery after AKI.^9^ In addition, despite tubular damage being the primary concern in AKI, only a few studies have begun to examine specific aspects of tubular health during and after AKI,^10^ the latter of which may provide insights to better link AKI to other long-term outcomes and help guide certain aspects of management.

In conclusion, the study of novel biomarkers has brought needed attention to critical phenotyping barriers that have hindered progress in AKI. The needed optimism of early adapters has undoubtedly advanced the field and provided a useful multifaceted framework that points to potential applications. Further work is needed to define specifically where biomarkers can help address uncertainties within current care to consolidate specific use cases and define the scope of utility for potential users. More critical advancements in utility will require tasking noninvasive biomarkers with the challenge of both confirming traditional and discovering novel AKI phenotypes. Success in these areas would reduce heterogeneity in clinical studies, help identify novel pathways and pathologic signatures, and provide a more comprehensive and accurate assessment of kidney health that remains long overdue.
